# Refinement of a Meaning-Centered Counseling Program for Chinese Patients with Advanced Cancer: Integrating Cultural Adaptation and Implementation Science Approaches

**DOI:** 10.21203/rs.3.rs-3576089/v1

**Published:** 2023-11-20

**Authors:** Florence Lui, Qingyi Zhang, George Bao, Bharat Narang, Ruo Yan Chen, Yunshan Niu, Jennifer Leng, William Breitbart

**Affiliations:** Memorial Sloan Kettering Cancer Center; Memorial Sloan Kettering Cancer Center; Weill Cornell Medicine; Memorial Sloan Kettering Cancer Center; Memorial Sloan Kettering Cancer Center; Memorial Sloan Kettering Cancer Center; Memorial Sloan Kettering Cancer Center; Memorial Sloan Kettering Cancer Center

**Keywords:** Asian Americans, cultural adaptation, implementation science, neoplasms

## Abstract

**Background:**

This mixed methods study identified needed refinements to a telehealth-delivered cultural and linguistic adaptation of Meaning-Centered Psychotherapy for Chinese patients with advanced cancer (MCP-Ch) to enhance acceptability, comprehensibility, and implementation of the intervention in usual care settings, guided by the Ecological Validity Model (EVM) and the Practical, Robust Implementation and Sustainability Model (PRISM).

**Methods:**

15 purposively sampled mental health professionals who work with Chinese cancer patients completed surveys providing Likert-scale ratings on acceptability and comprehensibility of MCP-Ch content (guided by the EVM) and pre-implementation factors (guided by PRISM), followed by semi-structured interviews. Survey data were descriptively summarized and linked to qualitative interview data. Three analysts independently coded the transcripts according to EVM and PRISM domains; discrepancies were resolved through discussion and consensus.

**Results:**

Quantitative findings showed high appropriateness and relevance of MCP-Ch across five EVM domains of Language, Metaphors/Stories, Goals, Content, and Concepts. Qualitative analysis yielded 23 inductive codes under the seven EVM domains: (1) Language (3 subcodes), (2) Persons (2 subcodes), (3) Metaphors/Stories (2 subcodes), (4) Methods (8 subcodes), (5) Content (2 subcodes), (6) Goals (4 subcodes), and (7) Concepts (2 subcodes). Themes based on PRISM included (1) Intervention characteristics (organizational perspective, 7 subcodes; and patient perspective, 6 subcodes) (2) External environment (2 subcodes), (3) Implementation and sustainability infrastructure (4 subcodes), and (4) Recipients (organizational characteristics, 5 subcodes; and patient characteristics, 4 subcodes).

**Conclusion:**

Recommendations for next steps include increasing the MCP-Ch protocol’s flexibility and adaptability to allow interventionists to flexibly tailor MCP-Ch material to meet patients’ individual needs, simplifying content to improve comprehension and acceptability, providing additional training to Chinese-serving providers to increase adoption and sustainability, and considering interpreter-assisted delivery to increase access. Findings yielded important information to maximize cultural relevance as well as the implementation and sustainability potential of MCP-Ch in real-world settings.

## Introduction

Asians are the fastest-growing racial/ethnic group in the United States [1]. The largest Asian subgroup is of Chinese origin, many of whom are foreign-born (76%) and limited English proficient (LEP) (48%) [2]. Cancer is the leading cause of death for both Chinese Americans (CAs) and Asian Americans overall [3]. CAs with cancer have a 22% higher mortality rate than non-Hispanic Whites (NHWs) with cancer after adjusting for age, stage at diagnosis and neighborhood deprivation [4], reflecting disparities in cancer screening, healthcare access, and health literacy [5, 6]. A population-based study examining factors associated with disparities in breast cancer survival among Asian women found that foreign-born Chinese women present with more advanced disease at diagnosis and, consequently, have lower survival rates than their U.S.-born counterparts, even after adjusting for age, socioeconomic status, and other factors [7].

Distress in Chinese patients with cancer is common, and distress risk is greater for patients with advanced disease: in one study, Chinese patients with advanced cancer had 1.85x the odds of reporting clinically significant distress than their earlier-stage counterparts [8]. LEP Chinese cancer patients are also more likely to report lower quality of life (QOL) [9] and untreated distress [10] than their English-proficient counterparts. In addition, the Chinese immigrant population may have unique sociocultural risk factors for cancer-related distress. For example, cancer fatalism (the belief that death is inevitable with cancer) [11] and cancer stigma (i.e., feeling rejected by others due to their cancer) [12, 13] can increase distress [14, 15].

Yet empirically supported mental health interventions developed for Chinese immigrants with advanced cancer are scarce. Our team’s systematic review and meta-analysis of psychosocial interventions for immigrant and LEP cancer survivors identified only five interventions that have been empirically tested for Chinese-speaking cancer patients [16–19], none of which were psychotherapy/counseling interventions [20]. There is a critical need to develop high-quality, culturally and linguistically tailored, and accessible mental health interventions for this growing yet underserved population [21].

## Meaning-Centered Psychotherapy

Meaning-Centered Psychotherapy (MCP) is a brief, structured, manualized intervention grounded in the work of Viktor Frankl [22]. MCP has demonstrated efficacy in enhancing patients’ sense of meaning-in-life and QOL while decreasing feelings of depression, anxiety, desire for hastened death, and distress in several randomized controlled trials [23–25]. MCP guides patients on how to access “sources of meaning” during challenging times: (1) historical (significant memories, relationships, and traditions and their impact on one’s sense of meaning), (2) attitudinal (confronting limitations imposed by cancer); (3) creative (engaging in life through one’s pursuits, including work, hobbies, life roles); and (4) experiential (connecting with life through experiences with love, nature, art, etc.).

MCP’s efficacy was tested in predominantly White samples and did not examine the impact of the intervention on Asians as a group [23–25]. Studies show culturally adapted treatments tailored for a specific group are 4x more effective than interventions for the general population [26]. Moreover, a growing body of research has demonstrated that spiritual well-being and a sense of meaning-in-life—key mediators of MCP treatment outcomes—are associated with higher QOL in Chinese cancer patients, suggesting meaning-centered interventions may be efficacious in this population [27–30]. Our formative research found that a meaning-centered intervention was relevant and acceptable to Chinese patients and their providers [31–33]. This work resulted in the initial development of a 6-session, telehealth-delivered MCP intervention manual for Chinese patients with advanced cancer (MCP-Ch) in Simplified Chinese and generated areas for additional inquiry, namely, the need to refine psychotherapeutic content to improve acceptability, comprehensibility, and relevance; and to identify factors that may affect implementation of MCP-Ch in usual care settings, including delivery via interpreters to increase access given the scarcity of bilingual providers [31].

## Bridging Cultural Adaptation and Implementation Science Models in Refining MCP-Ch

To meet our aim of reducing inequity in psycho-oncology care through integration of cultural factors in MCP-Ch and assessment of factors affecting uptake of MCP-Ch in usual care settings, the present study is guided by both cultural adaptation and implementation science. Both disciplines share the translational goals of advancing research into practice and increasing the accessibility of evidence-based interventions, but each employs a unique lens [34]. Cultural adaptation emphasizes maximizing an intervention’s acceptability and relevance by considering the cultural values, beliefs, and context of the population of interest [35], while implementation science aims to promote the adoption of evidence-based interventions and innovations into routine health care to improve population-level health [36]. Implementation science is therefore focused on multilevel contextual factors (e.g., consumer, provider, and organization), while cultural adaptation tends to address ethnocultural factors at the client or provider levels.

Integration between cultural adaptation and implementation science fields has been recommended to address the limitations of each [34]. The purpose of the present study was to further refine the MCP-Ch manual and protocol with feedback from Chinese-serving mental health professionals, with the goal of enhancing appropriateness and cultural relevance (guided by the Ecological Validity Model, EVM [35]) and maximizing uptake and sustainability in usual-care settings (guided by the Practical, Robust Implementation and Sustainability Model, PRISM [37]).

## Methods

### Description of MCP-Ch Intervention

Informed by prior formative research with Chinese patients with advanced cancer and their health providers [31–33], the initial MCP-Ch manual was culturally adapted for the Chinese cultural context and transcreated into Simplified Chinese for delivery in Mandarin Chinese, the most common dialect spoken by individuals of Chinese descent [38]. Initial adaptations included employing the “teach-back” method [39] to clarify abstract topics, adding resources on traditional Chinese medicine, nutrition information, and cancer myths and misconceptions, and shortening the number of sessions from seven to six [38] (Table 1).

### Research Frameworks

We utilized the Ecological Validity Model [35], a cultural adaptation framework, to gather stakeholder feedback on the MCP-Ch manual. Participants were asked to rate excerpts for cultural relevance and appropriateness across five of eight EVM domains: (1) Language (should be culturally syntonic); (2) Metaphors/Stories (i.e., sayings or stories utilized in the intervention, should be culturally relevant); (3) Content (i.e., how to handle cultural information about values, customs, and traditions in the intervention); (4) Concepts (i.e., concepts or theoretical constructs used in the intervention, should be culturally resonant); and (5) Goals (should be supportive of adaptive values from the patient’s culture). The (6) Persons (i.e., the role of ethnic/racial similarities and differences in shaping the patient-therapist relationship) and (7) Methods (i.e., procedures utilized in the intervention, should be culturally appropriate) domains were investigated through subsequent semi-structured interviews, in which participants discussed the intervention’s delivery methods and therapeutic strategies.

The Practical, Robust Implementation and Sustainability Model (PRISM) [37] framework was used to expand upon the EVM domain of (8) Context [35] to consider multilevel contextual factors from providers’ perspectives. We gathered stakeholder feedback on design choices that could facilitate MCP-Ch dissemination in usual-care settings, such as remote and interpreter-assisted delivery in the context of a shortage of bilingual providers. Survey items and semi-structured interview probes were guided by the 4 PRISM domains: (1) Intervention characteristics – organizational and patient perspective (e.g., Would their setting support use of MCP-Ch? Would the intervention appeal to patients?); (2) External environment (e.g., policies and regulations that might affect adoption and implementation); (3) Implementation and sustainability infrastructure (e.g., availability of resources); and (4) Recipients – organizational and patient characteristics (i.e., key characteristics of organizations and patients that might affect MCP-Ch uptake).

We followed the Standards for Reporting Qualitative Research (SRQR) [40] for reporting our methods and results.

### Participants and Procedures

Eligibility criteria were: (1) mental health provider; (2) fluency in spoken Mandarin/written Chinese; and (3) a minimum current or past caseload of three Chinese cancer patients. We used purposive sampling[41] to select 15 stakeholders based on their potential to provide valuable information based on their roles and expertise with the population of interest.

Following consent, participants were emailed a unique link to the study survey, which required approximately 60 minutes to complete. Subsequently, they participated in an online semi-structured interview. Interviews were conducted by a trained, bilingual clinical research coordinator (QZ) under the supervision of the study’s Principal Investigator (PI) (FL), from November 2022 to January 2023. Stakeholders were compensated with $50 upon survey completion and $50 upon interview completion. The Institutional Review Board at [redacted] approved all study procedures.

### Measures

#### Survey.

The survey collected information on 1) stakeholder demographics; 2) 3-point Likert-scale ratings (i.e., high, moderate, or low appropriateness/relevance) on EVM domains (i.e., Language, Metaphors/Stories, Content, Concepts, and Goals) for 22 key excerpts from the MCP-Ch manual; and 3) 5-point Likert-scale ratings ranging from “strongly disagree” to “strongly agree” on 15 statements describing MCP-Ch across the 4 PRISM domains (i.e., Intervention – organizational and patient perspective, External environment, Implementation and sustainability infrastructure, and Recipients – organizational and patient characteristics).

#### Semi-Structured Interview.

Interviews adhered to a semi-structured interview guide. Questions were guided by quantitative survey results and contained exploratory probes based on survey responses. The interviewer explicitly probed for items scoring “low” or “moderate” on the EVM domains, and “strongly disagree”, “disagree”, or “neutral” on PRISM domains (e.g., “You indicated the content in this section of the manual is ‘moderately appropriate’ in terms of cultural relevance. Could you please elaborate on what changes we could make to increase its relevance?”)

Interviews were conducted in Mandarin Chinese, except for one interview that was conducted in English and Chinese due to participant preference. Interviews ranged from 23 to 70 minutes. Interviews were audio recorded and transcribed by QZ, then translated into English by QZ or another trained Chinese linguist on the study team (YN). Interview transcripts in both Chinese and English underwent quality assurance for accuracy and were finalized after resolution of any discrepancies.

### Analyses

Survey data were analyzed using descriptive statistics. Qualitative data from interviews were analyzed using a hybrid deductive/inductive approach for EVM [42]. Deductive content analysis is a top-down process driven by a theoretical framework while inductive analysis is a bottom-up, data-driven process [43]. Specifically, seven of the EVM domains (i.e., Language, Persons, Metaphors/Stories, Methods, Content, Goals, Concepts) were selected a priori, with content coded according to these domains. Inductive themes were subsequently created to describe content in more detail and organized as subcodes of EVM domains. A deductive approach [44] was used to code content guided by PRISM domains; categories from the PRISM model were selected a priori, and themes were classified using the PRISM categories.

Five coders (FL, the PI and a clinical psychologist/researcher; QZ, the study’s Clinical Research Coordinator; RYC, a medical student; BN, a clinical research manager; and GB, a hospitalist/researcher) participated in the qualitative data analysis process. The PI (FL) provided training to coders prior to coding activities, including didactics about qualitative coding and an overview of the PRISM and EVM frameworks. Then, three coders independently coded the same transcripts. Intercoder reliability was established through team consensus building, facilitated by discussing points of divergence and convergence in team meetings moderated by the PI (FL). When divergence in the use of a code occurred, the team reviewed the transcripts, and coders provided their rationale for the use of the proposed codes. Discussions continued until the group reached a consensus on the codes selected and their application. Quotes to illustrate corresponding themes based on consensus meetings were selected by the PI (FL) and reviewed by the team.

## Results

### Survey Results

Characteristics of the sample are presented in Table 2.

Summaries of stakeholder ratings of MCP-Ch excerpts by EVM domain are reported in Table 3. A majority of stakeholders rated Language, Metaphors, Goals, Content, and Concepts as highly appropriate and/or relevant across all excerpts. However, Session 1 content was less favorably perceived, with 53.3% of stakeholders (*n* = 8) rating the Language and Concepts used in the Patient’s Definition of Meaning as having low to moderate appropriateness.

Stakeholders’ responses to the PRISM-guided pre-implementation survey are presented in Table 4. Most stakeholders (n = 13, 86.7%) were open to implementing MCP-Ch with Chinese patients with advanced cancer (item 1) and felt there was sufficient evidence to support a meaning-centered approach (item 2). All believed that Chinese patients with advanced cancer would find MCP-Ch helpful and acceptable (item 6). The majority (73.3%, n = 11) believed that telehealth delivery was safe and effective (item 4), and 80.0% (n = 12) considered it acceptable for Chinese patients with advanced cancer (item 7). The majority (60.0%, n = 9) believed remote delivery of interpreter-assisted interventions would be acceptable to Chinese-speaking patients (item 8). Over half perceived MCP-Ch as feasible for utilization in their practice setting (items 9 and 12). However, only 40% (n = 6) felt equipped with the necessary resources and training to apply MCP-Ch in their current practice setting (item 13).

### Interview Results by EVM

Hybrid deductive/inductive analysis identified 23 inductive codes under seven deductive/a priori EVM domains: (1) Language (3 subcodes), (2) Persons (2 subcodes), (3) Metaphors/Stories (2 subcodes), (4) Methods (8 subcodes), (5) Content (2 subcodes), (6) Goals (4 subcodes), and (7) Concepts (2 subcodes) (Table 5).

### Language

Stakeholders expressed the importance of using language in the MCP-Ch manual that was 1) plain and easy-to-understand: “*The expressions that you use right now are very formal, not colloquial. If you are talking to clients, you need to be more colloquial*.” (participant ID: 007). Stakeholders also gave suggestions to replace specific words and phrases with more 2) culturally syntonic language: “*‘Legacy’ may sound intimidating to your patients. You can say … ‘‘the continuation of your spirit*’ (participant ID: 003).” Finally, stakeholders recommended using 3) less directive language to introduce MCP-Ch concepts (e.g., meaning-in-life) to encourage patients to generate unique interpretations from their personal experiences: “*There doesn’t necessarily need to be a concrete definition of meaning. What matters the most for the patient is not the definition we give them, but what they value as the meaning of life themselves*” (participant ID: 004).

### Persons

Stakeholders highlighted the need to consider 1) patients’ region of origin and dialect, and 2) within-group differences among the larger Chinese population. Some stakeholders warned that the MCP-Ch manual, which was adapted from English to Mandarin Chinese based on language habits and preference in mainland China, might have limited applicability to Chinese patients from other regions, such as Hong Kong or Taiwan: “*I am bilingual in Mandarin and Cantonese. When I read this section based on the word order of Mandarin, I feel it flows pretty well, just like any books I read or listen to. But if it is delivered in Cantonese, I think it may be a little difficult to understand the way things are introduced*” (participant ID: 003). Additionally, stakeholders expressed that it was critically important for MCP-Ch counselors to be mindful of the significant within-group variability among the Chinese population: “*Since there’s a great deal of diversity among Chinese people, it’s important [for counselors] to be open-minded*” (participant ID: 002).”

### Metaphors/Stories

Stakeholders recommended that: 1) that stories and exercises be more specific and incorporate more detail: “*We can give specific examples when introducing a new point … you can use more examples from literature … or of some famous people who have cancer. Then you can dig deep into their stories. You can say, ‘Those are the things that represent the meaning of life for them. So, for you, what do you think is the meaning of life?*’” (participant ID: 013). In addition, they suggested 2) making stories/examples more culturally relevant: “*I think people may feel confused about [Viktor Frankl’s] name being brought up during the counseling session … It can be hard for the Chinese-speaking population to really understand the story of the concentration camp*” (participant ID: 003).”

### Methods

Stakeholders’ suggestions for refining MCP-Ch intervention methods included 1) providing more orientation to the MCP-Ch program to highlight the program’s structure, goals, and target population: “*If I were the patient, what could I gain from this program? Should I be more hopeful in life?... This [program goal] doesn’t seem very clear to me*” (participant ID: 005). In addition, stakeholders recommended 2) addressing potential distress, 3) using a more active approach, 4) providing culturally appropriate education about cancer, 5) integrating peer support into the program, and 6) the importance of therapeutic alliance/relationship building. Stakeholders also discussed the use of 7) interpreters and 8) remote delivery. While some providers expressed reservations about the use of interpreters, many acknowledged its strength to increase access to mental health care: “*If the alternative is no treatment, I think this [delivering therapy through an interpreter] is still much better than nothing ... It’s really difficult, but it doesn’t necessarily mean that it cannot be done well*” (participant ID: 014). A few providers were similarly apprehensive about the effectiveness of remote delivery among older and more vulnerable patients. However, a majority felt its advantages (i.e., increasing access to care) outweighed these disadvantages.

### Content

Stakeholders suggested refining MCP-Ch’s cultural content by: 1) addressing religious beliefs, which could significantly influence one’s understanding of various MCP-Ch concepts (e.g., transcendence, something greater, and meaning): “*For people who affiliate themselves with Christianity or other religious groups, ‘something greater’ may mean ‘higher power’, right? For atheistic individuals, ‘something greater’ may mean ‘the universe’. Anyway, I think we should explain to them [the patient] the meaning of ‘something greater’. Some patients coming from mainland China have religious affiliations and others do not*” (participant ID: 002) Stakeholders also recommended that the manual be refined to highlight 2) family-centered sources of meaning: “*Most Chinese families heavily emphasize a sense of family and relationships*” (participant ID: 014). “

### Goals

Stakeholders discussed how 1) taboos around discussing EoL could affect treatment goals: “*They may not be willing to use that word [“legacy”]. Patients may already know that they are dying, but they may not be willing to think about death or start end-of-life planning*” (participant ID: 015). Stakeholders also discussed the need to tailor therapeutic goals based on level of 2) prognostic awareness. Many Chinese families adopt a family-centered model of care (in contrast to a Western patient autonomy model), whereby the cancer diagnosis and prognosis is delivered to a designated family member rather than to the patient directly. To protect the patient from suffering emotional distress, some families opt to withhold the cancer diagnosis entirely or provide only limited information to the patient: “*a lot of family members don’t want the patients to know their illnesses, and they don’t want the patients to know that they are dying. Even if the patients have a tumor, their family doesn’t tell them the tumor has metastasized*” (participant ID: 012).

Stakeholders also shared that 3) meaning-centered interventions were especially needed in contemporary society because “*immigrants bury themselves in their jobs, building a better future for their children*” (participant ID: 014). Finally, they emphasized the 4) need to incorporate promoting hope and acceptance as a part of therapeutic goals: “*Even when they are dying, they are leaving with hope, not despair… We want our patients to feel like, ‘[even if] I lost the battle, it was a fair fight*’” (participant ID: 005).

### Concepts

Stakeholders highlighted 1) specific MCP concepts and materials that were too difficult to understand: “*It’s hard to connect with ‘creativity,’ maybe it’s a bit abstract, very philosophical*” (participant ID: 001). In addition, providers noted 2) the need to clarify or draw connections between MCP concepts: “*Creativity is related to responsibilities. We are talking about creativity, courage, and commitment, right? These are all related to the roles that a person has in society, which are also related to their experiences and life limitations in the past, present, and future. These ideas are all connected*” (participant ID: 009).

### Interview Results by PRISM

Themes were deductively coded according to a priori categories within four PRISM domains: (1) Intervention characteristics – organizational perspective (7 subcodes) and patient perspective (6 subcodes), (2) External environment (2 subcodes), (3) Implementation and sustainability infrastructure (4 subcodes), and (4) Recipients – organizational characteristics (5 subcodes) and patient characteristics (4 subcodes). Table 6 (please see Table 6 attachment) presents the coded themes, illustrative quotes, and relevant pre-implementation factors by PRISM domains.

### Intervention Characteristics

#### Organizational perspective.

*Readiness* (n = 3) pertained to stakeholder assessments of whether providers’ usual care setting was equipped to initiate an intervention like MCP-Ch. S*trengths of the evidence base* (n = 4) reflected 1) uncertainty about effectiveness of remote counseling and 2) the need for evidence of efficacy of the MCP-Ch adaptation. *Addressing barriers of frontline staff* (n = 2) concerned addressing uncertainty about employing remote modalities and managing risk in an advanced cancer context, as well as implementation barriers for frontline staff due to high patient turnover in the palliative care setting. *Coordination across departments and specialties* (n = 2) highlighted the need for collaboration between social work, psychiatry, and oncology departments. Examples of themes about *burden (complexity and cost)* (n = 2) included the administrative burden of implementing a research project in a usual care setting and the time burden involved in potentially using consecutive (versus simultaneous) interpreters in delivering MCP-Ch to LEP patients. The importance of MCP-Ch’s *usability and adaptability* (n = 3) was reflected in comments about the need to flexibly tailor the intervention to each patient: “*People may have different cultural, familial, or educational backgrounds. So you may need to be flexible … modify it while doing it*” (participant ID: 006). Stakeholders also described the need to observe clinically significant improvements in their patients (*ability to observe results*, n = 2).

#### Patient perspective.

*Patient centeredness* (n = 10) themes included the importance of 1) centering patient’s experiences in MCP-Ch delivery (e.g., medical experience, past trauma, and life history), and 2) evaluating appropriateness for remote delivery on a case-by-case basis. *Providing patient choices* (n = 3) included allowing the patient to assign their own definitions to meaning-centered concepts. Stakeholders recommended revising the manual to address low literacy and socioeconomic status as barriers to care (*addressing patient barriers*, n = 8): “*For patients with lower education levels, they might not know how to answer your questions since the content is very abstract*” (participant ID: 013).

Comments about the *seamlessness of transition between program elements* (n = 6) focused on the merits of simultaneous vs. consecutive interpretation in improving seamlessness of MCP-Ch delivery: *“[Consecutive] interpretation will interrupt the flow. When the patient is crying, and you say, ‘Wait a minute, we’ll need to interpret this,’ ... this would greatly impede the natural flow of the patient’s thoughts and emotions*” (participant ID: 006). Themes about *service and access* (n = 5) concerned 1) trade-offs involved in increasing access via interpreters, 2) the need to integrate psychological care with oncology care, and 3) the use of telehealth delivery to increase access. Participant *burden (complexity and cost)* (n = 5) comprised 1) time burden, especially in the context of multiple appointments and cancer treatment, 2) the complexity of involving interpreters, and 3) the complexity of MCP’s existential underpinnings.

### External Environment

Comments about the *regulatory environment* (n = 4) concerned telehealth regulations as a potential barrier to implementation: “*There are related laws and regulations [for telehealth] … You need to justify the reason and prove that confidentiality and privacy are well protected. What should you do if a patient is suddenly under some physical emergency during a video meeting? There will be regulations related to this matter*” (participant ID: 009). Stakeholders’ discussion of *community resources* (n = 2) involved suggestions to partner with local, national, and international Chinese-serving organizations to enhance dissemination of MCP-Ch.

### Implementation and Sustainability Infrastructure

Stakeholders discussed the need for a credentialed “point person” to establish the MCP-Ch program in their work setting (*dedicated team*, n = 2). Comments concerning *adopter training and support* (n = 5) included the need for training on 1) mental health, 2) cultural competency, and 3) meaning-centered interventions.

Regarding *relationship and communication with adopters (bridge researchers)* (n = 3), stakeholders observed that mutually beneficial partnerships could facilitate implementation and sustainability of MCP-Ch: “*I think if we really want to carry it out, both parties must fully understand each other … Also, we hope to see that the results of our partnership will be beneficial for both sides*” (participant ID: 013). Comments related to *adaptable protocols and procedures* (n = 2) included the 1) applicability of MCP-Ch to other cancer populations, including early-stage and pediatric; 2) adaptability of MCP-Ch to group delivery and 3) potential to add a peer support element to the intervention.

### Recipients

#### Organizational characteristics.

*Organizational health and culture* (n = 2) involved 1) growing recognition of the importance of mental health in cancer and 2) the need to consider the “bottom line” due to the profit-driven culture of many healthcare organizations: “*This decision [to implement the program] involves the financial interest of the company … if your program can bring more revenue to the hospital*” (participant ID: 013). Regarding *management support and communication* (n = 2), stakeholders emphasized the necessity of achieving “buy-in” from colleagues at higher levels within one’s organization. Comments regarding *shared goals and cooperation* (n = 1) focused on the need to establish mutual goals with Chinese-serving hospitals and clinics. Stakeholders’ comments about *systems and training* (n = 3) concerned the need for organizations to offer more training on psycho-oncology/mental health, working with Chinese patients, working with a cancer population, and working with interpreters. With regards to *staffing and incentives* (n = 5), comments concerned evaluations of staff capacity and the potential for non-mental health professionals (e.g., nurses) to be trained in and deliver MCP-Ch, given a national shortage in trained mental health professionals.

#### Patient characteristics.

Stakeholders discussed the need to consider patients’ *demographics* (n = 6), including age, literacy, socioeconomic status, cultural background, and region of origin. Comments about *disease burden* (n = 6) concerned the cognitive, emotional, and physical burdens associated with advanced cancer. *Competing demands* (n = 2) included patients’ work and family demands, which could present a barrier to participation in MCP-Ch: “*When someone already has very limited time, would he be willing to spend so much time on [counseling]? That’s also something to consider*” (participant ID: 006). Regarding patients’ *knowledge and beliefs* (n = 7), stakeholders recognized the following barriers to MCP-Ch uptake: 1) respect for authority figures/doctors as a potential block to developing an authentic therapeutic relationship, 2) unfamiliarity with counseling, 3) concerns about confidentiality, and 4) limited prognostic awareness and related EoL taboos: “*Chinese people are usually conservative, and don’t want to talk about life and death*” (participant ID: 012).

## Discussion

The results of the present mixed-methods study indicated that while MCP-Ch material, content, and approaches were considered by stakeholders to be broadly relevant and acceptable in meeting the needs of Chinese patients with advanced cancer, the MCP-Ch manual and protocol would benefit from refinements to improve cultural relevance and comprehensibility along EVM dimensions and to account for multilevel contextual factors along PRISM domains to facilitate implementation in usual-care settings.

Findings structured by the EVM yielded important insights into areas of the MCP-Ch manual that required further refinement. Within the Language, Persons, and Concepts domains, stakeholders suggested that some patients might have difficulty understanding complex MCP terminology. They also suggested using less directive language to define MCP-Ch concepts and instead allow patients to generate their own interpretations. Within the Methods domain, stakeholders discussed how patients unfamiliar with counseling would benefit from additional orientation to the MCP-Ch intervention and counseling more generally. Within the Content domain, stakeholders highlighted the importance of family-centered sources of meaning given Chinese culture’s collectivistic emphasis on family relationships. Under Goals, stakeholders discussed cultural taboos around discussing death and EoL, suggesting that intervention content be tailored based on patients’ prognostic awareness and readiness and willingness to engage in EoL discussions.

The PRISM-guided findings similarly generated important insights into potential barriers to and facilitators of MCP-Ch adoption and uptake in usual care settings. For example, stakeholders observed that thorough consideration of a patient’s *demographics, disease burden*, and culturally-based *knowledge and beliefs* could increase the MCP-Ch intervention’s *patient-centeredness*. Therefore, maximizing MCP-Ch’s *usability and adaptability* (i.e., by allowing counselors to flexibly tailor the material to meet an individual patient’s needs) would improve program implementation and uptake. Notably, all stakeholders who commented on the use of interpreters preferred simultaneous to consecutive interpreting due to a perception that the former would better facilitate therapeutic alliance and save time. Indeed, prior studies have shown that simultaneous medical interpreting resulted in fewer errors, more utterances, and higher patient satisfaction than consecutive methods [45–47]. Many highlighted *adopter training and support* as a key facilitator of MCP-Ch implementation. Developing a comprehensive model of training that includes supervision, education about meaning-centered approaches and the specific adaptations made to MCP for the Chinese cancer population, access to remote training materials, and content on telehealth delivery and how to work effectively with interpreters could address this implementation need. Moreover, engaging in promotion and partnership with *community resources* (i.e., Chinese-serving organizations)—e.g., by sharing resources on MCP-Ch—could aid in increasing referrals while building a mutually beneficial *relationship with adopters*.

In light of these findings, our next steps for MCP-Ch includes the following to enhance ecological validity per EVM and facilitate implementation per PRISM:

1. Increase flexibility and adaptability of MCP-Ch intervention: The revised intervention manual will include cultural adaptation notes with instructions on how to modify or tailor content and delivery methods to address low literacy; cancer stigma; diversity in region of origin, socioeconomic status, religion, etc. (EVM: Persons, Methods, Concepts; PRISM: Intervention Characteristics, Recipients) and different prognoses/levels of prognostic awareness (EVM: Goals, PRISM: Recipients). To meet stakeholders’ usability needs, an avenue for future research could include developing a transdiagnostic MCP-Ch manual to include Chinese-speaking survivors across all stages, given the dearth of psycho-oncology interventions for Chinese patients, and a brief (i.e., 3-session) version [48] to reduce time burden (PRISM: Intervention Characteristics).
2. Simplify content to improve comprehension and acceptability and to reduce patient burden and intervention complexity: The revised manual will use plain language that is easily understood across all literacy levels, and all take-home exercises will be simplified and revised. Plain language sample scripts will be provided for the counselor (EVM: Language, PRISM: Intervention Characteristics). Stories and exercises will be revised to increase cultural relevance (EVM: Metaphors/Stories, Concepts; PRISM: Intervention Characteristics).
3. Provide additional orientation: To reduce mental health stigma and introduce patients to a potentially culturally unfamiliar practice, the first session will include information about counseling and why it may be needed during the cancer experience (EVM: Methods, PRISM: Recipients).
4. Intervention implementation through telehealth: MCP-Ch was designed to be telehealth-delivered to increase access and address barriers to uptake (e.g., disease burden, transportation barriers, and competing demands). Interventionists will receive training on how to conduct MCP-Ch via telehealth, including how to navigate patient distress and conduct risk and safety assessments remotely (EVM: Methods, PRISM: Intervention Characteristics, Recipients).
5. Adopter (i.e., interventionist) training support and supervision: We will develop a virtual asynchronous interventionist training that includes background on psycho-oncology and the needs of Chinese cancer patients, information about MCP-Ch and the cultural adaptation process, and remote supervision. Other future plans include the development of a website to aggregate resources (e.g., training videos, readings) to facilitate dissemination of MCP-Ch beyond the research study to practitioners at usual-care settings (EVM: Methods; PRISM: Implementation and Sustainability Infrastructure).
6. Intervention implementation through remote medical interpreters to increase access: In addition to training bilingual providers on the MCP-Ch program, we are partnering with the Language Initiatives team within the Immigrant Health & Cancer Disparities Service at MSK—which addresses language access needs—to enable interpreter-assisted MCP-Ch delivery. An English-version of the culturally adapted MCP-Ch (Simplified Chinese) manual will facilitate MCP-Ch delivery by English-speaking providers via English-Chinese interpreters, to be pilot-tested in a future trial. Given stakeholder concerns about the use of interpreters and the cultural competencies of English-speaking counselors, we will partner with the Language Initiatives team to provide a training for interventionists on how to work effectively with interpreters (EVM: Methods, Persons; PRISM: Intervention Characteristics, External Environment, Implementation and Sustainability Infrastructure).
7. Interpreter training support to build capacity: The Language Initiatives team has developed a robust and interactive virtual interpreter training program for Chinese-English interpreters led by YN. The 80-hour training covers professional standards of practice and ethics, medical terminology, and linguistic/cultural responsiveness, and a mandatory exam to certify interpreters meet professional interpreter standards. We will partner with local, minority-serving academic institutions to identify and recruit bilingual students to receive this training to increase capacity (PRISM: Implementation and Sustainability Infrastructure).

Limitations of our study include a mixed method design (i.e., surveys followed by semi-structured interviews) that may not be replicable or applicable to other interventions. In our semi-structured interviews, we probed only survey responses that reflected low to moderate acceptability, relevance, or appropriateness. This was a time-saving measure meant to elicit refinements to the MCP-Ch intervention manual and protocol but may have skewed the results toward statements of what is “wrong” rather than what already “works.” Although we elicited patient and other stakeholder perspectives (health care providers including oncologists, nurses, etc.) in prior formative work that informed the initial MCP-Ch adaptation [31–33], we recruited only mental health providers for the present study. Inclusion of additional stakeholders could have yielded additional insights.

## Conclusions

Quantitative and qualitative findings yielded suggestions for refinement to reduce barriers to MCP-Ch uptake by addressing cultural, contextual, and systems-level factors in the adapted intervention. The present study represents an illustrative example of how to bridge cultural adaptation and implementation science [34] in intervention adaptation, development, and planning.

## Figures and Tables

**Table 1:** Overview of MCP-Ch Sessions

Session # and Topic	Session Description
1) MCP concepts & overview	Introduce MCP key concepts; patients share their cancer stories
2) Cancer & identity	Patients explore how their sense of identity has been affected by cancer
3) Meaning from your personal story	Patients reflect on past experiences, current accomplishments, and legacy they wish to pass on
4) Attitude & meaning/End-of-life	Patients explore their attitudes toward limitations imposed by cancer
5) Meaning via creativity & experience	Patients connect with meaning derived from creativity, courage and responsibility; and from their experiences of love, beauty, and humor
6) Transitions	Reflections and hopes for the future

**Table 2:** Characteristics of Mental Health Providers (N = 15)

Characteristic	n (%)
Sex	
Male	3 (20.0)
Female	12 (80.0)
Ethnicity	
Chinese	15 (100.0)
Birthplace	
Mainland China	13 (86.7)
Taiwan	1 (6.7)
Hong Kong	1 (6.7)
Preferred Language	
English	2 (13.3)
Mandarin	11 (73.3)
Cantonese	1 (6.7)
English or Chinese	1 (6.7)
Primary Profession	
Mental health professional	11 (73.3)
Health professional	1 (6.7)
Palliative care professional	1 (6.7)
Researcher	1 (6.7)
Other	1 (6.7)
Practice Location	
United States	8 (53.3)
China	4 (26.7)
Canada	2 (13.3)
Taiwan	1 (6.7)
Academic Degree	
Masters	11 (73.3)
PhD or PsyD	2 (13.3)
MD	1 (6.7)
Other	1 (6.7)
Years in Clinical Practice	
2 or less	4 (26.7)
3–5	3 (20.0)
6–10	4 (26.7)
11–15	1 (6.7)
15 or more	3 (20.0)
Weekly number of Chinese cancer patients	
Less than 5	9 (60.0)
5–10	4 (26.7)
11–20	1 (6.7)
21–30	1 (6.7)
Weekly number of Chinese advanced cancer patients	
Less than 5	10 (66.7)
5–10	4 (26.7)
11–20	1 (6.7)

Percentages may not equal 100% due to rounding.

**Table 3:** Provider Responses to MCP-Ch Manual Excerpts by Ecological Validity Model Domain (N = 15)

		*Language Appropriateness*		*Metaphors/Stories Appropriateness*		*Goals Relevance*		*Content Appropriateness/Relevance*		*Concepts Appropriateness/Relevance*	
		*L/M*	*H*	*L/M*	*H*	*L/M*	*H*	*L/M*	*H*	*L/M*	*H*
*Session*	*Content*	n (%)	n (%)	n (%)	n (%)	n (%)	n (%)	n (%)	n (%)	n (%)	n (%)
1	Intervention Introduction	7 (46.7)	8 (53.3)	4 (26.7)	11 (73.3)	6 (40.0)	9 (60.0)	6 (40.0)	9 (60.0)	3 (20.0)	12 (80.0)
1	Patient’s Cancer History	5 (33.3)	10 (66.7)	1 (6.7)	14 (93.3)	1 (6.7)	14 (93.3)	2 (13.3)	13 (86.7)	2 (13.3)	13 (86.7)
1	Patient’s Definition of Meaning	8 (53.3)	7 (46.7)	4 (26.7)	11 (73.3)	2 (13.3)	13 (86.7)	3 (20.0)	12 (80.0)	8 (53.3)	7 (46.7)
1	Overview of Session Topics	4 (26.7)	11 (73.3)	0 (0.0)	15 (100)	1 (6.7)	14 (93.3)	0 (0.0)	15 (100)	4 (26.7)	11 (73.3)
1	Introduction to Viktor Frankl	2 (13.3)	13 (86.7)	1 (6.7)	14 (93.3)	1 (6.7)	14 (93.3)	2 (13.3)	13 (86.7)	2 (13.3)	13 (86.7)
1	What Do We Mean By “Meaning”?	3 (20.0)	12 (80.0)	1 (6.7)	14 (93.3)	0 (0.0)	15 (100)	2 (13.3)	13 (86.7)	5 (33.3)	10 (66.7)
1	Session 1 Exercise	3 (20.0)	12 (80.0)	0 (0.0)	15 (100)	0 (0.0)	15 (100)	0 (0.0)	15 (100)	0 (0.0)	15 (100)
2	Introduction to Session 2	3 (20.0)	12 (80.0)	0 (0.0)	15 (100)	0 (0.0)	15 (100)	0 (0.0)	15 (100)	0 (0.0)	15 (100)
2	Session 2 Exercise	3 (20.0)	12 (80.0)	1 (6.7)	14 (93.3)	0 (0.0)	15 (100)	0 (0.0)	15 (100)	2 (13.3)	13 (86.7)
2	Invite Family Member/Caregiver	1 (6.7)	14 (93.3)	0 (0.0)	15 (100)	1 (6.7)	14 (93.3)	0 (0.0)	15 (100)	1 (6.7)	14 (93.3)
3	Introduction to Session 3	5 (33.3)	10 (66.7)	0 (0.0)	15 (100)	0 (0.0)	15 (100)	0 (0.0)	15 (100)	4 (26.7)	11 (73.3)
3	Meaning in Our Past	1 (6.7)	14 (93.3)	0 (0.0)	15 (100)	0 (0.0)	15 (100)	0 (0.0)	15 (100)	0 (0.0)	15 (100)
3	Meaning in Our Present and Future	4 (26.7)	11 (73.3)	0 (0.0)	15 (100)	0 (0.0)	15 (100)	2 (13.3)	13 (86.7)	0 (0.0)	15 (100)
4	Introduction to Session 4	4 (26.7)	11 (73.3)	0 (0.0)	15 (100)	1 (6.7)	14 (93.3)	0 (0.0)	15 (100)	2 (13.3)	13 (86.7)
4	Confronting Life’s Limitations	5 (33.3)	10 (66.7)	1 (6.7)	14 (93.3)	0 (0.0)	15 (100)	0 (0.0)	15 (100)	1 (6.7)	14 (93.3)
4	“Legacy Project”	2 (13.3)	13 (86.7)	0 (0.0)	15 (100)	0 (0.0)	15 (100)	1 (6.7)	14 (93.3)	2 (13.3)	13 (86.7)
5	Introduction to Session 5	4 (26.7)	11 (73.3)	1 (6.7)	14 (93.3)	0 (0.0)	15 (100)	1 (6.7)	14 (93.3)	1 (6.7)	14 (93.3)
5	Meaning Through Creativity	3 (20)	12 (80.0)	1 (6.7)	14 (93.3)	0 (0.0)	15 (100)	0 (0.0)	15 (100)	2 (13.3)	13 (86.7)
5	Meaning Through Experience	2 (13.3)	13 (86.7)	2 (13.3)	13 (86.7)	1 (6.7)	14 (93.3)	0 (0.0)	15 (100)	2 (13.3)	13 (86.7)
6	Process Transitions	1 (6.7)	14 (93.3)	0 (0.0)	15 (100)	0 (0.0)	15 (100)	0 (0.0)	15 (100)	0 (0.0)	15 (100)
6	Legacy Project Review	0 (0.0)	15 (100)	0 (0.0)	15 (100)	0 (0.0)	15 (100)	2 (13.3)	13 (86.7)	1 (6.7)	14 (93.3)
6	Feedback about MCP-Ch	0 (0.0)	15 (100)	0 (0.0)	15 (100)	0 (0.0)	15 (100)	0 (0.0)	15 (100)	0 (0.0)	15 (100)

H, high appropriateness or relevance; L/M, low-to-moderate appropriateness or relevance

**Table 4:** Provider Responses to PRISM Pre-Implementation Assessment (N = 15)

		*SD/D*	*Neutral*	*A/SA*
*Item*	*Item Statement*	n (%)	n (%)	n (%)
1	I would use MCP-Ch in my work with Chinese patients with advanced cancer	0 (0.0)	2 (13.3)	13 (86.7)
2	I believe there is a good evidence to support a meaning-centered approach to meet the psychosocial needs of Chinese patients with advanced cancer	0 (0.0)	2 (13.3)	13 (86.7)
3	My theoretical background and training lends itself well to using MCP-Ch with patients	0 (0.0)	1 (6.7)	14 (93.3)
4	I believe telehealth-delivered mental health interventions are safe and effective	1 (6.7)	3 (20.0)	11 (73.3)
5	I believe mental health interventions such as MCP-Ch can be delivered remotely to Chinese-speaking patients by monolingual English-speaking therapists via interpreters	5 (33.3)	3 (20.0)	7 (46.7)
6	Based upon my experience with the patient population, I believe Chinese patients with advanced cancer would find MCP-Ch helpful and acceptable	0 (0.0)	0 (0.0)	15 (100)
7	Based upon my experience with the patient population, I believe Chinese patients with advanced cancer would find telehealth delivery acceptable	1 (6.7)	2 (13.3)	12 (80.0)
8	Based upon my experience with the patient population, I believe Chinese patients with advanced cancer would find an interpreter-assisted, remotely delivered counseling program acceptable	4 (26.7)	2 (13.3)	9 (60.0)
9	MCP-Ch could be feasibly utilized in my practice setting	1 (6.7)	4 (26.7)	10 (66.7)
10	My supervisor/administration would support my use of MCP-Ch	1 (6.7)	4 (26.7)	10 (66.7)
11	My patients’ psychosocial needs (accounting for patient characteristics such as income, education level, acculturation level, cancer type, and current medical and/or mental health treatment) would be well met by MCP-Ch	1 (6.7)	1 (6.7)	13 (86.7)
12	Implementing MCP-Ch in my current practice setting is feasible	0 (0.0)	6 (40.0)	9 (60.0)
13	I have the resources and training I need to use MCP-Ch in my current practice setting	3 (20)	6 (40.0)	6 (40.0)
14	The current state and federal regulatory environment is conducive to delivering MCP-Ch via telehealth	0 (0.0)	6 (40.0)	9 (60.0)
15	The policies of my practice setting do not conflict with my use of MCP-Ch in clinical practice	2 (13.3)	3 (20.0)	10 (66.7)

SD/D, strongly disagree or disagree; A/SA, agree or strongly agree

**Table 5:** Codes, Illustrative Quotes, and MCP-Ch Refinements by Ecological Validity Model (EVM) Domain

EVM Domain	Codes Associated with EVM Domain	Illustrative Quotes [participant ID]	MCP-Ch Adaptations/Refinements by Code
Language	Use less directive language	There doesn’t necessarily need to be a concrete definition of meaning. What matters the most for the patient is not the definition we give them, but what they value as the meaning of life themselves. [004] Each person may have a different interpretation of the meaning of life. I feel like this should be an open discussion, conveying the idea of acceptance and inclusion to the patient. [006] In terms of the general structure of the manual, you often say, “this is how we defined it, what are your thoughts?” It feels like the manual is telling me that 1 need to know your definition, even if my definition and your definition might not be the same ... I think there are approaches that might work better. For example, you can say, “this is your thought and this is ours, may we reach a compromise?” ... I am not sure about how to ask it in a better way, but right now the manual makes me feel like the client has to agree with what the counselor says. [008]	Replace “teach-back” method with “think aloud” approach for discussions of key MCP terminology
	Use plain, easy-to-understand language	It’s kind of not so user-friendly for the professionals ... Because some of the wording, even though understandable, when it is used [by] the professionals, it will become very, what do you say, very formal, yeah. It is very formal. [001] If you come up to them and start saying words that sound fancy, first, it’s possible that the patients don’t really understand what those words mean. Second, they might feel a little offended - “why are you asking me questions using words that I don’t understand?” ... So, what I am saying is, try to use relatively approachable language or everyday expressions [002] Being easy to understand is a must. [006] If you are talking to clients, you need to be more colloquial. This will help you get closer to them, especially because they usually have high levels of anxiety and fear. [007] If [patients] have a lower literacy level and you don’t ask the question in a way that’s easy to understand, you won’t be able to get much out of them. [012]	Simplify language, e.g., replace “meaning” with meaning-in-life, “transcendence” with “going beyond oneself”, “limitations” with “barriers”
	Use culturally syntonic language	So the order of Chinese expression here is more of an English way of expression, and it’s not a way of Chinese expression. So then, I changed the order of the words. So when you read them, they sound more Chinese. [001] I don’t usually use “learnings” or “lessons”. For Chinese people, “learning” is something you pass down when you do something good, like when you win an award. It’s something happy. But you won’t really use this word [in the cancer context]. [007] Even though sometimes you and the client are using the exact same word, such as “value”, you may actually mean different things, right? For example, when you talk about “tradition,” the client may understand the word “tradition” in a completely different way. Some Chinese people would definitely think of it as traditional festivals. They won’t be thinking about family tradition or family values. [007]	Improve sentence flow and word choices, e.g., replace “lessons” with “what you have learned”, “homework” with “take-home exercises”
Persons	Consider region of origin and dialect	Cantonese people still speak Mandarin ... but there are certain vocabularies ... [This may work better for] ... these Mandarin-speaking Chinese cultural groups. [001] When I read this section based on the word order of Mandarin, I feel it flows pretty well, just like any books I read or listen to. But if it is delivered in Cantonese, I think it may be a little difficult to understand the way things are introduced. [003] Personally, I am from mainland China, but I know that people from mainland China, Taiwan, Hongkong and Macao may talk in different ways. So I’m curious about the target audience, and whether you’d consider a simplified version or a traditional Chinese version. Because even though the content is similar, participants may not have the same language preferences. [008]	Add cultural adaptation note with examples of how to use self-disclosure to aid the working alliance (e.g., by acknowledging commonalities and/or differences when they apply, such as being a fellow migrant/from a family of immigrants, or sharing the same language)
	Be aware of within-group variability	Since there’s also a great deal of diversity among Chinese people, it’s also important [for counselors] be open-minded. For counselors who are culturally competent, they may serve the Chinese groups with more flexibility, considering the diversity within the Chinese populations. [002] I think this is also very different from person to person. Sometimes how we should say a word depends on the patient’s level of education, or their mental status. I think there may be several different words we can use interchangeably. The patient’s situation will decide which word is more appropriate. [006]	Add cultural adaptation notes throughout manual to orient counselors to within-group variability
Metaphors/Stories	Be more specific/use more detail in stories and exercises	Basically, you often need to provide very concrete examples - “okay, what have changed in your life since your diagnosis?” You wanted to ask about limitations and losses, right? You can say, “is there anything you used to do freely that has become difficult for you now? If so, can you give me an example?” [002] I think it’s better to include more details, instead of being very general about it ... By digging deeper into it, the participant can see the logic more clearly. Then, he will be able to self-reflect after the counseling session, or when they are not in the session. [004] My understanding of this story is that there is an immigrant named Zhang Wei who came from his hometown to the US to pursue a new life, but he was diagnosed with advanced cancer. Even though he really wanted to go home, he was not able to. However, by keeping close contact with his family, he could still feel the love and the meaning of life. He could still express love to his family. He still remembers his favorite dish from his hometown, or something like that. Or, he thinks about the rice dumplings his grandma made. Well, my understanding is that you need to make the participant feel connected to his experiences. Even though he’s far away from where he wants to be, he could still feel connected to these experiences. Then, he will find meaning in life. This is how I thinkyou can explain this story further and be more clear. [008] I think for patients with lower education levels, they might not know how to answer your questions since the content is very abstract. We can give specific examples when introducing a new point ... you can use more examples from literature. I mentioned the book “The Temple of Earth and Me” written by the Chinese writer Shi Tiesheng. Or you can use the examples of some famous people who have succeeded in combatting cancer. Then you can dig deep into their stories. You can say, “Those are the things that represent the meaning of life for them. So, for you, what do you think is the meaning of life?” [013]	Add more detail to stories and metaphors in manual, e.g., the story of the immigrant Zhang Wei; add concrete examples to experiential exercises, e.g., the exercise on love, beauty, and humor
	Make stories/examples more culturally relevant	In the Chinese culture, for people who are not Buddhists, Christians, or Catholics, they may think of “Supreme Deity ()” as “something greaterthan oneself.” What I am saying is that some people may know what you mean by “something greaterthan oneself” right away, but not everyone can understand this concept. For those who don’t understand it, we can provide some examples, right? Some people may be familiar with the concept of “the will of the Supreme Deity ()”, right? I’m not sure how much detail you can include in the manual, but I think it’s important to find ways to explain the concept of “something greaterthan oneself” to those patients who don’t understand it. I think it refers to some form of “higher power,” right? Right, then we should explain to them what “higher power” means, right? I am thinking that there may be a counterpart in Chinese, like “the will of the Supreme Deity, ().” This may be more comprehensible to Chinese patients. [002] Sometimes when you are talking about examples involving parents and kids, you could also take into consideration their grandparents, since grandparents are sometimes more involved in a Chinese family. [008] The example you are using now [Frankl] is based on foreign literature, and the character is a foreigner. But your program is targeting Chinese people. That’s why I think the example you are using, which is Frankl’s experience in the concentration camp, is not in line with what you mentioned earlier ... [Consider] adding] some examples related to the Chinese culture. [013]	Add culturally syntonic stories/examples and expressions to illustrate MCP concepts, e.g., using the story of a well-known Chinese historian Sima Qian to illustrate “he who has a *why* to live for can bear almost any how”
Methods	Provide orientation to MCP-Ch program	[Patients] will have questions about what kind of program it is, and they may have different understandings of what the program is ... there are many kinds of programs. It could be an activity, a workshop, a research study. So I think we need to explain a little bit here. [001] Participants will be more confident if the counselor could organize the plan with them, instead of creating goals that are too ambitious to achieve. The plan can be very detailed, even including the things that can be done on that day. By doing so, the participants will feel more empowered, and as if things are not so difficult in the end. [004] I think it can be stated more clearly that this program is set up for patients with advanced cancer ... You can mention at the beginning that we have learnt from past experience that a majority of advanced cancer patients will experience some loss of meaning or have some anxiety about life. And this is what this program is set to help with. I think I’ll add a lot of normalizing language first, and then frame it. We can ... say less about counseling. For example, don’t say, “I want to heal you,” but rather say, “if you’re interested, I can help you explore this together. We understand that meaning exploration helps many people cope with this very difficult time.” [008] A large part of the Chinese culture is about our connection with our family. Some Chinese patients might not consider participating in such projects if they have a good relationship with their families. You could also mention in the beginning that some of the content of our discussion may be things that we don’t normally share with people outside of the family, and we understand that it might not be something they’re comfortable with in the beginning. [008]	Refine program introduction to orient the patient to the idea of counseling and discussing potentially private matters with a counselor
	Address potential distress	I think this topic [roles and responsibilities] may trigger their emotions because they may no longer be able to commit to work, or to handle the responsibilities as a parent. I’m just saying that this topic may be a trigger for them. This topic is certainly important, but you may need to consider this possibility of their emotions being triggered during the counseling process ... when you train the counselors, it may be necessary to prepare them for handling this kind of situation if it does come up. [003] You need to be more alert when exploring trauma remotely. Since most of your topics are meaning centered, if they fall into very deep and sad emotions, you may need to ... look out for that. [007]	Add cultural adaptation notes on suggestions for working with patients who are distressed, e.g., emphasize the fact that the patient survived difficult experiences etc.
	Consider using a more active approach	I want to put more emphasis on what can be done now and in the future. This can motivate the participants to practice on their own when they leave the session. What they have learned in the session should not be something that only exists in their imagination. They can have a to-do list for the day or for the week, which can better motivate them to act. [004] We can [lead] them ... [to show how] meaning emerges. The patient may say, “The reason I’ve been working so hard is because I saw my parents working very hard. That’s why I think...” Then, I would say, “Oh, so you’ve inherited this value from your parents. That’s why you’ve kept on working hard.” And then, after we tell them about this legacy, we can say, “Actually when you’re working hard, you’re also influencing your friends at the same time. You have created something unintentionally. Though you don’t know the meaning of your work, you may hear feedback from other people around you. This is a kind of gain as well.” We can give some examples like this to illustrate that meaning can emerge from experiences. [009]	Add a list of questions as a guide for patients to share their life story with others
	Provide culturally appropriate education about cancer	Another difficult reality is that some doctors do not like it when I talk about traditional Chinese medicine concepts. But there are also those who are supportive of traditional medicine. Some medical experts invite traditional Chinese medicine practitioners to their lectures. [005]	Include resources on traditional Chinese medicine, cancer facts and common misconceptions
	Importance of therapeutic alliance/relationship building	But first, the therapist needs to understand the cancer patients better ... If the therapist can get in touch and understand the patient first, instead of just forcing our knowledge onto them, the process will be more natural. [004] Even if we speak the same language, the quality of the meeting still depends on a lot of other factors, such as if you can understand clearly what your patient is talking about. It’s also about the therapist’s attitude. If you can continue to build a relationship with the patient, you may still overcome the language limitation. [009] I will give them a lot of validations. I will try to understand them, understand why they can’t process those positive things. I will validate and try to understand their feelings and tell them that those feelings are normal. Only when they have been accepted, heard, supported, and cared about, will they start to feel like, “Okay, life is not that terrible after all...at least my counselor is here with me and listening to me.” [011]	Emphasize the role of a therapists as a learning partner and a witness to the patient’s life story
	Use of interpreters	I object to delivering MCP via interpreters, because I always believe that it’s not simply a language barrier issue. It’s also a cultural competency issue. As we discussed before, I don’t believe someone without cultural competency can explain clearly what the sense of meaning and purpose means ...Additionally, explaining these concepts in culturally relevant ways is not the job of the interpreters. Interpreters only interpret what the counselors have said word by word, so they can’t serve the role of cultural clarifier. [002] But if the alternative is no treatment, I think this [delivering therapy through an interpreter] is still much better than nothing ... It’s really difficult, but it doesn’t necessarily mean that it cannot be done well ... The dynamics will be changed by the presence of the interpreter ... However, there are definitely ways to work with this dynamic. For example, there are some training courses on how to perform treatments with the assistance of an interpreter. [014]	Provide training for both interpreters and interventionists; discuss role of interpreter in initial session and in discussions about patient-counselor confidentiality
	Remote delivery	I think telehealth interventions may be difficult for Chinese patients. Most of them prefer in-person interventions. Especially for patients who are more sensitive and vulnerable, they may hope that you can give them a hug, hand them a tissue, or at least be physically present. They may feel more comfortable this way. Also, counselors can observe the patient’s current status more directly. [003] I do a lot of remote work. Especially when the clients are in different places, it’s sometimes very difficult to meet with them face-to-face .... I think you need to assess the appropriateness case by case. Especially for those whose conditions are very serious, for example, those with suicidal ideations, telehealth-delivered interventions might not be safe and effective for them. [011] Maybe more people have gotten more used to telehealth after COVID-19. Telehealth is also very convenient. It provides an option for people who have trouble moving around. [015]	Provide orientation on telehealth delivery
	Provide peer support	When people with the same condition come together, the strength of the group will motivate individuals to share ... It is very important for patients to inspire, comfort, and heal each other. [007] I also think that it would be better to incorporate peer support in the program. Patients do not just seethe counselor in one-one-one sessions, but also meet other cancer patients. [015]	n/a, consider adding peer support element in future iterations
Content	Address religious beliefs	Some Chinese Christian patients might feel like God has slowly left them since they got cancer. Or, in the past, the patient was a very firm believer, but since they have gotten sick, they felt like “God has gradually left me”. But... of course you would only hear things like this from the Christian or the Catholic community. I think for most Chinse patients, if they don’t believe in those two religions, it might be difficult for them to feel what you describe here. They might not know what prayers are. Is it a wish, or is it some hope for changes in life? [004] Personally, I don’t have any religious beliefs, but I think when exploring the meaning of life, this is a very important aspect to consider ... I think “karma” is a very important concept, and people with different religious beliefs will have different understandings ... I would suggest having at least a note in the manual, saying that if the participant has religious beliefs, they could talk about their religious beliefs’ impact on their understanding of the meaning of life. [008]	Add cultural adaptation notes to orient therapists when introducing concepts like “transcendence” and “something greater than oneself”
	Address role of family	During the visit, one patient told me that he thought he was useless because he wasn’t able to do anything and had always been a burden to the family. Then I would say, “Indeed, your disease is very difficult, but if you have a positive mindset, that would be your contribution to your family.” Even after we pass away, our family members might still face other difficulties, and they might also feel anxious and worried at some point. However, if they can think about their mom or dad or whomever and recall how brave and calm they were when facing such difficulties in life, they would feel encouraged to face any challenges in their own lives. That’s how you contribute to your family ... How they face this critical challenge of life is one contribution they can bring to others. [005] In the literal sense, meaning is about what you have left for the world, right? But for some clients, meaning is about whether they have left something for their families and children ... A lot of Chinese people feel that they don’t have enough time to say goodbye or grieve for their loved ones. Especially when they talk about the deaths of their parents, it is usually very touching, because they are facing the same thing now themselves. [007] A large part of the Chinese culture is about our connection with our family. Some Chinese patients might not consider participating in such projects if they have a good relationship with their families. [008] Why do some people fulfill their responsibilities? Because they live for their family. They want to fulfill their responsibilities. That’s because of their love. But when some family members fulfill the responsibilities to take care of the patient, they may not do it out of love. They may do it just fortheir own peace of mind. So, you will see that some people aren’t really willing to take care of their family because they don’t have strong love for their family. On the other hand, some patients themselves want to die. They want to die because of their love for others. They don’t want to be a burden for their family. [012]	Add cultural adaptation notes to emphasize the importance of considering collectivistic cultural values; add examples/stories that center on responsibility and love for the family
Goals	Taboos around discussing death/end-of-life planning	Because in my experience, I found that for many cancer patients, especially when they have not yet completed their first six cycles of chemotherapy, maybe they’re only at the second or the third cycle, they would not want to use the word “cancer”, they are a bit resistant about this. They would just say, “Oh, I have a disease”, or “there is something wrong with my body.” So I think it’s a bit psychologically unacceptable to raise the question of whether their cancer can be cured or not before the patient has completed all six cycles of chemotherapy. [004] So, some people may be very sensitive when they hear the word legacy because Chinese people are usually conservative, and don’t want to talk about life and death. [012] I just think that mentioning “legacy” to Chinese people when they are still alive is kind of like a taboo. They may not be willing to use that word. Patients may already know that they are dying, but they may not be willing to think about death or start end-of-life planning. Or, they may be more concerned about financial legacies. “Spiritual wealth” may be a bit more euphemistic ... Legacy makes it sound like this session is about inheritance planning. Legacy may not be appropriate since some people are very sensitive. [015]	Ask the patient about how they would like to be remembered or what they wish to leave behind instead of using the term “legacy”
	Differences in prognostic awareness	I’m not sure about your patients, but over here, a lot of family members don’t want the patients to know their illnesses, and they don’t want the patients to know that they are dying. Even if the patients have a tumor, their family don’t tell them that the tumor has metastasized ... Based on the patients’ conditions, the answers they give you may be different. The answers from people who know they are sick are drastically different from those who don’t know how their illnesses may impact their lives ... So, you need to adjust your plan based on the patients’ condition ... Make your therapeutic goal relevant to patients’ situations. Otherwise, you may only get an “official” answer from the patients because they just want to be polite. [012]	Add cultural adaptation notes on how to work with patients differently based on their prognostic awareness
	Need for meaning in contemporary Chinese culture	Nowadays, generally speaking, there is a lack of the sense of meaning ... in the generation of our grandparents, everything they did was connected. The place you worked was also where your kids went to school, where you took a shower, where you lived with your good friends. It all took place in a dormitory. These things were all connected and concrete. The farmers ate what they grew. They knew where the meal was coming from. But in our current society, there is no such thing anymore ... Based on my observation, most Chinese immigrants bury themselves in their jobs, in their efforts to live a so-called good and happy life, and in building a better future for their children. These become their meaning. But suddenly when their bodies are faced with this existential struggle, many of their previous sources of meaning are suspended. Then it’s easy forthem to start contemplating the questions of “why I’ve been living this way?” [014] I think “meaning” may not have been adequately addressed in this population. Most people think about planning for financial inheritances ... Most people prioritize survival, including how to fight cancer, how to feel better, and how to manage pain. Not many people think about spiritual legacy. I think it’s also helpful to provide opportunities for them to gain a sense of closure. [015]	n/a
	Promote hope/acceptance	I think we need to adjust and change the way we interact with them and deliver hopeful messages to them. Even when you are dying, you are leaving with hope, not despair. [005] Yes, I think we can incorporate acceptance. But of course, making a change will be the best-case scenario, right? Changes from negative to positive, that is definitely the best. But then, they need to accept it first, and then slowly change their attitude into a positive one based on their own pace, right? [007] In my view, as a counselor, we definitely want to make the clients feel like, “Oh, I am still doing good.” We want to help them find hope and the meaning of life during this very difficult period of time, instead of making them feel like everything is dark and hopeless. From the perspective of counselors, we need to bring our clients hope. [011]	Establish the connection between “transcendence” and “acceptance”
Concepts	Some MCP concepts/material difficult to understand	It’s hard to connect with “creativity”, or maybe it’s a bit abstract, very philosophical. It doesn’t connect with their daily life in a practical sense ... So Viktor Frankl’s theory is very philosophical by itself. However, we have to express it in a practical way, so that the audience can get what you are trying to ask, and they can answer your questions, telling you what they think about meaning in their minds. [001] And then, I think people may feel confused about the author’s name being brought up during the counseling session. Based on the Chinese population I have worked with before and their educational backgrounds, I think they may feel confused when they hear a long name like Viktor Frankl. [003] I would actually focus more on letting them know what it feels like to have a sense of meaning. When a person has a sense of meaning, he would feel fulfilled, right? Feeling relieved, right? Although fear is still there, we have our inner strength. These are all brought by meaning. So I think it’s better not to use words like greatness and transcendence. [007] I think the concept of meaning can be very broad, but if you change it to the meaning of life, it may be a little easier for people to understand. [008]	Add concrete examples and metaphors; recommend “think aloud” approach to elicit associations the patients have made to the material
	Clarify/draw connections between MCP concepts	Every session has a theme, but these themes are all related to each other. So, when you explain it in the very first session, you should convey this idea first. You need to make it clear to the patient about why we are doing ... You have 4 sources of meaning: meaning from what you've inherited, from attitudes, from creativity and experience. There are connections among these 4 sources. You need to let the patient know why we need to talk about legacy and attitude towards life limitations, and why we need to talk about creativity. Creativity is related to responsibilities. We are talking about creativity, courage, and commitment, right? These are all related to the roles that a person has in society, which are also related to their experiences and life limitations in the past, present, and future. These ideas are all connected. [009] Your legacy project is also about creating things, like writing journals and making a photo album ... this legacy project can be a part of creativity, or it can be derived from creativity. This way, the patient will be clear about the relationship between those two things. [013]	Add cultural adaptation notes to emphasize the difference between the creative and experiential source of meaning

**Table 6.** Codes, Illustrative Quotes, and Pre-Implementation Factors by Practical, Robust Implementation and Sustainability Model (PRISM)

PRISM Categories	Providers endorsing n (%)	Illustrative Quotes [participant ID]	Pre-implementation factors
***Intervention Characteristics – Organizational perspective***			
Readiness	3 (20%)	Our hospital’s service for cancer patients mainly focuses on providing resources ...Our hospital may not [offer therapeutic services] for the time being ... Instead, we would refer patients [elsewhere]. [001]	Whether usual care setting is prepared/set up to offer counseling services
Strength of the evidence base	4 (27%)	The effectiveness of telehealth counseling is not that good. Based on my reading, telehealth counseling is not very effective compared to in-person counseling. [003] I think this program is very meaningful and I recognize its value. I think [implementing it at my workplace] mainly depends on the efficacy and feasibility of this program [011]	Uncertainty about effectiveness of remote counseling Need for evidence of efficacy of MCP-Ch adaptation
Addresses barriers of frontline staff	2 (13%)	Counselors would be really worried if their patients get upset and leave in the middle of the sessions. The counselors won’t know if their patients are safe or not. [003] We have to do home visits. We have new cases coming in and patients passing away. So there’s high patient turnover. Every two or three days, there may be patients who passed away. So if we haven’t established the program, and patient has passed away, then, it will be very difficult for the program to be implemented. [006]	Concerns about using remote modality and managing risk in this context Implementation barriers for frontline staff - competing responsibilities in the context of high patient turnover
Coordination across departments and specialties	2 (13%)	The social work team itself may have some resources for this, but for oncology cancer patients, we don’t have a specific social worker. For the departments that implement it, we will need to ... see how we can cooperate in terms of staffing and scheduling. [001] We mainly involve nurses in performing psychological interventions because doctors usually work in outpatient clinics and nurses are in the wards. We have nurses specialized in mental health or psychiatry to participate in this. There needs to be a collaboration between the psychiatry and oncology department. [013]	Need to coordinate with social work team and other departments Need for collaboration between psychiatry and oncology departments
Burden (complexity and cost)	2 (13%)	When you implement this program, you have to recruit patients, right? When you recruit patients, of course you need to go through a lot of IRB processes, consent, and all these things. These may all become concerns on the hospital’s side. [001] The intervention may take longer. There may be some cultural differences as well. Overall, not very efficient ... I think the simultaneous medical interpretation should be great. This way, the intervention won’t take more than 6 weeks to complete. [015]	Administrative burden of implementing a research project in a usual care setting Time burden involved in using consecutive (vs. simultaneous) interpreters in delivering an intervention to LEP pts
Usability and adaptability	3 (20%)	Having personalized information would be most helpful for these patients because everyone’s meaning of life varies considerably. I think the topics in the sessions should be personalized in nature. I may have my unique meaning of life that is different from yours. [002] Such great material ... But then, I also think that people may have different cultural, familial, or educational backgrounds. So, often times, you may need to be flexible ... modify it while doing it. [006]	Ability to modify/flexibly tailor material based on pt’s individual needs
Trialability and reversibility	0 (0%)		
Ability to observe results	2 (13%)	For the departments that implement it, we will need to seethe value of this resource. We can see if there’s an opportunity to try it out. [001] [My practice setting] would need to see whether such training is conducive to improving the quality of my work. [009]	Need for organization to observe results and show value
**Intervention Characteristics – Patient perspective**			
Patient centeredness	10 (67%)	I think for therapists, they need to get in touch and understand the patient first ... For example, what kind of medical procedures they will go through and what kind of physical changes they will have ... [then] the process will be more natural. [002] It’s important to evaluate them case by case. Some terminal cancer patients may have trauma. [004] I think it is important for the interpreter to know the patient, especially in this journey of meaning exploration. [007] You need to evaluate the appropriateness [for remote delivery] case by case. [If] it’s difficult for them to stay engaged online, or that their conditions are more serious, I would suggest combined remote and in-person sessions for them. [013]	Center pt’s experiences, including medical experiences, trauma, and life history Evaluate appropriateness for remote delivery on case-by-case basis
Provides patient choices	3 (20%)	Each person may have a different interpretation of the meaning of life. I feel like this should be an open discussion, conveying the idea of acceptance and inclusion to the patient. [006] Another point is that I think you can give your patients a chance to test it [remotely delivered via an interpreter] out. They may think that this delivery method works just fine after the first session. [015]	Importance of allowing patient choices re: interpreting MCP concepts Give patient choice/opportunity to test out interpreter/remote delivery modality
Addresses patient barriers	8 (53%)	If you are talking to [low SES] clients, you need to be more colloquial. In fact, even for clients with higher education levels, it’s still better to be colloquial. This will help you get closer to them, especially because they usually have high levels of anxiety and fear. [007] [Patients with advanced cancer] are already in great suffering. If they have a lower literacy level and you don’t ask the question in a way that’s easy to understand, you won’t be able to get much out of them. [012]	Need to address low literacy and SES as barriers
Seamlessness of transition between program elements	6 (40%)	[With consecutive interpreting], it feels like the talk is on and off, right? After you say one or two sentences, you wait for the interpreter to interpret. I think this doesn’t help Chinese-speaking patients share their stories. I think simultaneous interpreting is definitely better than consecutive interpreting, although the dynamic of the session might be different. [008] It would be ideal if you could have one interpreter work with the same counselor consistently. If the counselor has to work with a different interpreter for each session, that will be really weird. [015]	Consecutive vs. simultaneous interpretation in facilitating seamless delivery of intervention
Service and access	5 (33%)	Of course, we should use the assistance of interpreters if we don’t have resources for them otherwise. Delivering the interventions remotely by monolingual English-speaking therapists is still viable, but I think the effectiveness of the interventions may be compromised, compared to in-person delivery. [002] Cancer patients go to outpatient clinics at hospitals very often to receive examinations or long-term treatments. I think it may be more appropriate if the counselors can offer these intervention sessions at the hospitals where the patients receive examinations and treatments. [003] Yes, I truly think [providing counseling via interpreters] will work. I have done it myself, for example, providing therapy for Spanish-speaking patients, and providing treatment for patients who speak minority languages. It is a little more difficult than working with patients who speak Chinese or English. But if the alternative is no treatment, I think this is still much better than nothing.... It’s really difficult, but it doesn’t necessarily mean that it cannot be done well. [014] I think there are many benefits [to telehealth-delivered MCP]. One is to provide access to those with advanced cancer who are not even able to get out of bed. I think this is very valuable. Many people are not able to go in-person due to various other reasons. For example, it’s inconvenient for people with compromised immune system to leave their homes. [014]	Tradeoffs involved in increasing access via interpreters Need to integrate psychological care with oncology care Use of telehealth delivery to increase access
Burden (complexity and cost)	5 (33%)	Based on my understanding of Chinese patients with advanced cancer, they really value their time. If this intervention is to be implemented in my work setting, I expect it to be when the patients visit the hospital to receive other medical services. I don’t think they will have much motivation if they have to come to the hospital just to receive the counseling service. [003] Patients may be distracted by having to focus on making sense of what the interpreters have said. This is difficult, too. The intervention may take longer. There may be some cultural differences as well. Overall, not very efficient. However, if you aren’t able to provide bilingual services, offering this intervention via interpreters is better than no intervention at all. That’s for sure. [012] Those patients have been in great torture and anxiety for a long time. They also have great despair facing death ... How much energy do the patients have to think about these [existential] questions? I don’t think this is practical enough. [012]	Time burden Concerns about complexity of involving interpreters Concerns about complexity of program
Feedback of results	0 (0%)		
**External Environment**			
Payor satisfaction	0 (0%)		
Competition	0 (0%)		
Regulatory environment	4 (27%)	I think it depends on the states and their regulations. ... License transfers among different states might pose some restrictions. For example, you have a New York license but want to practice in California, or you have a California license but want to practice in Illinois. [004] There are related laws and regulations [for telehealth] ... You need to justify the reason and prove that confidentiality and privacy are well protected. What should you do if a patient is suddenly under some physical emergency during a video meeting? ... And then, as for devices for telehealth, how should hospitals and other clinical settings set up these devices? There will be regulations related to this matter. [009]	Telehealth regulations as a potential barrier to implementation
Reimbursement	0 (0%)		
Community resources	2 (13%)	We could look at where Chinese patients would go for resources .... These resources are very good, and they should be available in places where there are a lot of Chinese patients. [004]	Partner with Chinese-serving organizations
**Implementation and Sustainability Infrastructure**			
Performance data	0 (0%)		
Dedicated team	2 (13%)	If we want to do this, it’s almost certain that we need to appoint a person to take care of this project. And that person also needs to be trained in mental health. [001]	Need for “point person” to establish the program in their work setting
Adopter training and support	5 (33%)	More importantly, if you wish to implement this program, the hospital will need to hire and train specialized [mental health] professional staff. [003] In terms of training, the hospital where I previously worked provided a lot of training for therapists and social workers. Nevertheless, the training was mostly based on Western culture. There wasn’t much training specifically targeting the Chinese-speaking population. I think it would be better if you can provide one or two days of professional training ... specifically for the Chinese-speaking population. [003] If your program’s interventionists can offer some [MCP training] support for us, we may be able to establish partnerships. I think nurses from these two psychiatry and oncology] departments can be trained together as a whole. 013]	Need for mental health training Need for training on working with Chinese-speaking population Need for MCP training
Relationship and communication with adopters (bridge researchers)	3 (20%)	We have an oncology ward at our hospital. We also carry out some psychotherapy programs there. But you guys [specialize in] psychological counseling ... We may be able to establish some partnership. We also feel that it may be more convenient to carry out the program online. ... I think if we really want to carry it out, both parties must fully understand each other. For example, we also need to know about your team’s qualifications. And then we can talk about cooperation ... Also, we hope to see that the results of our partnership will be beneficial for both sides. I’m saying that we hope to see good results that can be beneficial to both your side and our side so that we can keep the program going. [013]	Mutually beneficial partnerships can facilitate implementation and sustainability of an intervention
Adaptable protocols and procedures	2 (13%)	In fact, some children suffer from cancer too ... Children can also find the meaning of their lives and see what they can do during the last days of their life. This is the same with adults. In fact, if you can take good care of the children during their last periods of life, their families will feel very relieved. This is very touching and meaningful. So I also think we can expand the target population to children and teenagers. You can also think about group sessions. [007] I think this intervention may also be helpful to patients with early-stage cancer ... There are a lot of patients with early-stage cancer ... I also think that it would be betterto incorporate peer support in the program. Patients do not just see the counselor in one-one-one sessions, but also meet other cancer patients. They can communicate with each other. I think group-based sessions may be more efficient. [015]	Applicability of MCP to other cancer populations, including early-stage and pediatric; adaptable to group delivery and addition of peer support
Facilitation of sharing of best practices	0 (0%)		
Plan for sustainability	0 (0%)		
**Recipients – Organizational Characteristics**			
Organizational health and culture	2 (13%)	This decision [to implement the program] involves the financial interest of the company. The management team makes their decisions based on practical considerations, such as ... if your program can bring more revenue to the hospital. [003] In recent years, more and more doctors recognize the importance of mental health for cancer patients. ...They started taking mental health more seriously. [005]	Profit-driven culture of health organizations Importance of treating mental health of cancer patients
Management support and communication	2 (13%)	The most important thing would be to get the approval from the head of the higher-level department. If we try pushing it this way, it might be easierto implement. [001] I am not a decision-maker. I am a social worker. To implement this program, you need to discuss with personnel from different admin levels on its possibilities, development, and potential. [006]	Need for buy-in from “higher ups”
Shared goals and cooperation	1 (7%)	I think it would be fine if you partner with hospitals that have established services for the Chinese population and implement the program through them. [003]	Shared goals with Chinese-serving hospitals
Clinical leadership	0 (0%)		
Systems and training	3 (20%)	There isn’t much training specifically targeting the Chinese-speaking population. [003] I think the interpreters should also have some knowledge about psychology and should be able to properly empathize with the patients ... they should also convey the therapist’s tone and emotions ... I think for therapists, they need to understand these cancer patients first. For example, what kind of medical procedures they will go through and what kind of physical changes they will have. [004] For example, there are some training courses on how to perform treatments with the assistance of an interpreter ... about how to modify the dynamics and what to do when there is one more person in the session, including the seating arrangement for the three people in in-person sessions. How to set up the meeting, and to inform the interpreter about the situation of the meeting? How to set up the interpreter so that the interpreter can better facilitate the session? How to set up the patient to ease the potential anxiety or adjust their physical distances? These are the many ways to work with these dynamics. [014]	Need for more training on psycho-oncology/mental health services, working with Chinese patients, about cancer, and on working with interpreters
Data and decision support	0 (0%)		
Staffing and incentives	5 (33%)	It’s important to consider if the hospital has enough staff to implement this counseling program and if the staff have enough capacity. My previous work setting didn’t possess these characteristics because they were very short of staff. Over there, even though counselors and oncology social workers served the function of providing counseling sessions, they usually had too many patients. They could have more than 30 cases to deal with each day and were also involved in various kinds of activities. They wouldn’t have time to provide this type of structured counseling. [003] Secondly, you need to evaluate how much time is needed, how many staff you need, and how much time they need to spend on it besides their regular work schedule. I don’t think the training itself is difficult. Rather, it’s about whether we have the capacity to do it. [006] We have specialized nurses in different fields in each hospital. So, we have nurses who are specialized in mental health. I think it can also be counted as one of our resources. [013]	Need for more staff/evaluation of capacity; potential to use nurses trained in mental health
Expectation of sustainability	0 (0%)		
**Recipients – Patient Characteristics**			
Demographics	6 (40%)	Especially if they are, I assume they are of certain age for cancer with late stage, not necessarily but most of the time, they are older age people. And in terms of their cognitive or some other physical situations, you should make it as easy to understand as possible. [001] Yes, because most of my patients at the time were with lower literacy, education, and income levels. They were unfamiliar with counseling ... I am worried that they won’t be able to understand a lot of concepts. [003] I also think that people may have different cultural, familial, or educational backgrounds. So, often times, you may need to be flexible. [006] Consider different language habits ... Personally, I am from mainland China, but I know that people from mainland China, Taiwan, Hongkong and Macao may talk in different ways ... Because even though the content is similar, participants may not have the same language preferences. [008]	Geriatric, literacy, socioeconomic status, cultural backgrounds, region of origin
Disease burden	6 (40%)	Especially for patients who are seriously ill and undergoing different types of treatments, their ability to concentrate may not be as good as usual. They may have very sharp minds in general, but their attention won’t be able to last long during this special time. Thus, I think it may be very exhausting for them to read these scripts consisting of very long sentences. [003] For example, there might be memory losses and response limitations. There might be distinct good days or bad days at the beginning that are highly unpredictable. [004] Some people may feel very painful and wouldn’t be able to talk to you for more than five minutes. Some people may not be physically in pain, but emotionally they are aware that they are in the advanced stage, and ... their moods are also very very low. [006]	Cognitive, emotional, and physical burdens of advanced cancer
Competing demands	2 (13%)	When someone already has very limited time, would he be willing to spend so much time on [psychotherapy]? That’s also something to consider. He may feel, ‘I have very little and limited time right now, I’d rather ... I’m already in poor health. I’ve been sleeping all day. For the few minutes, hours I’m awake, I may want to spend that time with my family.’ [006] Based on my understanding of Chinese people, they might have devoted themselves to work. [008]	Work and family demands
Knowledge and beliefs	7 (47%)	Our Chinese patients are all very polite. If they hear something they don’t understand, they may just not let you know. Or, if they don’t really want to have a deep conversation with you, they would still be polite and let you know that they don’t have this need. This is a common situation. [002] For Chinese people at this stage in their life, I’m not sure how much they will be interested in exploring meaning. I simply think that they may not be that interested. If the patients already have advanced cancer, they may wonder, “Why would I participate in such a counseling program? I’m already at the end of my life and I have no interest in exploring my meaning.” Most of my patients at the time were with lower literacy, education, and income levels. They were unfamiliar with counseling. [003] [Chinese patients] might be worried that the interpreter might not keep the information confidential and share the session information with the patient’s family or other Chinese people. [008] A lot of family members don’t want the patients to know their illnesses, and they don’t want the patients to know that they are dying. Even if the patients have a tumor, their family don’t tell them that the tumor has metastasized. ... Chinese people are usually conservative, and don’t want to talk about life and death. [012] I think “meaning” may not have been adequately addressed in this population. Most people think about planning for financial inheritances. Not many people think about spiritual legacy. I think it’s also helpful to provide opportunities for them to gain a sense of closure. For example, let them write letters to their close ones in order to help them gain a sense of closure [015]	Hierarchy/respect for doctors as potential block to therapeutic relationship Unfamiliarity with counseling Concerns about confidentiality Prognostic awareness/family-centered model of care Need for meaning-centered approach

## Data Availability

The datasets used and/or analyzed during the current study are available from the corresponding author on reasonable request.
